# Antimalarial Activity of Aqueous Extracts of Nasturtium (*Tropaeolum majus* L.) and Benzyl Isothiocyanate

**DOI:** 10.3390/molecules29102316

**Published:** 2024-05-15

**Authors:** Ana Maria Pintão, Tiago Santos, Fátima Nogueira

**Affiliations:** 1 Egas Moniz School of Health & Science, University Campus, Quinta da Granja Monte da Caparica, 2829-511 Caparica, Portugal; 2 Egas Moniz Center for Interdisciplinary Research (CiiEM), Egas Moniz School of Health & Science, University Campus, Quinta da Granja Monte da Caparica, 2829-511 Caparica, Portugal; 3 Instituto de Higiene e Medicina Tropical (IHMT), Universidade NOVA de Lisboa, Rua da Junqueira 100, 1349-008 Lisboa, Portugal; a21001315@ihmt.unl.pt (T.S.); fnogueira@ihmt.unl.pt (F.N.); 4 Global Health and Tropical Medicine (GHTM), Associate Laboratory in Translation and Innovation Towards Global Health, LA-REAL, Instituto de Higiene e Medicina Tropical, IHMT, Universidade NOVA de Lisboa, UNL, Rua da Junqueira 100, 1349-008 Lisboa, Portugal; 5 LAQV-REQUIMTE, MolSyn, IHMT, Universidade NOVA de Lisboa, UNL, Rua da Junqueira 100, 1349-008 Lisboa, Portugal

**Keywords:** antimalarials, *Plasmodium falciparum*, *Tropaeolum majus* L., benzyl isothiocyanate

## Abstract

Malaria remains an important and challenging infectious disease, and novel antimalarials are required. Benzyl isothiocyanate (BITC), the main breakdown product of benzyl glucosinolate, is present in all parts of *Tropaeolum majus* L. (*T. majus*) and has antibacterial and antiparasitic activities. To our knowledge, there is no information on the effects of BITC against malaria. The present study evaluates the antimalarial activity of aqueous extracts of BITC and *T. majus* seeds, leaves, and stems. We used flow cytometry to calculate the growth inhibition (GI) percentage of the extracts and BITC against unsynchronized cultures of the chloroquine-susceptible *Plasmodium falciparum* 3D7 − GFP strain. Extracts and/or compounds with at least 70% GI were validated by IC50 estimation against *P. falciparum* 3D7 − GFP and Dd2 (chloroquine-resistant strain) unsynchronized cultures by flow cytometry, and the resistance index (RI) was determined. *T. majus* aqueous extracts showed some antimalarial activity that was higher in seeds than in leaves or stems. BITC’s GI was comparable to chloroquine’s. BITC’s IC50 was similar in both strains; thus, a cross-resistance absence with aminoquinolines was found (RI < 1). BITC presented features that could open new avenues for malaria drug discovery.

## 1. Introduction

Antimalarial drug development remains strongly linked to plant-based pharmaceuticals, as some of the most important therapeutics are based on their chemical scaffolds, such as aminoquinolines (e.g., chloroquine) and endoperoxides, i.e., artemisinin-based drugs [1,2]. The main causative agent of malaria, *P. falciparum*, has developed resistance to all antimalarial drugs in clinical use, including aminoquinolines and endoperoxides [3,4,5,6]. Furthermore, the development of these antimalarials involves unaffordable environmental and economic costs for most malaria-endemic countries, hence the WHO’s encouragement of applying natural extracts or plant-based pharmaceuticals based on traditional medicines [7,8]. Therefore, we propose a strategy that aims to repurpose traditional medicinal plants for antimalarial applications.

*Tropaeolum majus* L. (*T. majus*), an herbaceous plant commonly known as garden nasturtium, belongs to the family *Tropaeolaceae*, and it is native to Peru [9,10] It was first introduced to Europe in the sixteenth century and then spread to other parts of the world, including malaria-endemic countries, such as Angola, Rwanda, and Vietnam [9,10,11]. *T. majus* was selected as a potential candidate for antimalarial drug development, not only because of its widespread distribution but also because of its traditional usages against bacterial infections, such as bronchitis, sinusitis, and urinary tract infections, as well as for its antifungal and antiviral activities [12,13,14].

The broad therapeutic spectrum of *T. majus* can be linked to a group of compounds known as glucosinolates [15,16,17,18]. Glucosinolates are stable water-soluble precursors of isothiocyanates located in vacuoles [19]. When plant tissues are damaged, the endogenous enzyme myrosinase (Thioglucoside hydrolase, EC 3.2.3.1), which is stored in myrosinase grains of the myrosin cells, is released and combined with glucosinolates to produce biologically active compounds, such as nitriles, thiocyanates, isothiocyanates, epithionitriles, and oxazolidine-2-thiones [19]. The degradation products formed depend on physiological conditions such as the pH and the presence of cofactors. Isothiocyanates are formed at a neutral pH, while at acidic pH, nitriles are the dominant products [18].

The benzyl glucosinolate metabolite is found in every part of the *T. majus* plant, especially in the seeds [15,16,17,18,20]. When hydrolyzed by the endogenous enzyme myrosinase (Thioglucoside hydrolase, EC 3.2.3.1), it generates a variety of breakdown products, including benzyl isothiocyanate [19]. BITC (C8H7NS), with a molecular weight of 149.21 g/mol, is lipophilic and poorly soluble in water [21]. It is the sole isothiocyanate present in *T. majus*, and in mammals, BITC is metabolized in the liver via degradation and conjugation by glutathione-S-transferase and glutamyl transpeptidase, respectively [21]. It is reported that 62% of BITC is excreted in urine as mercapturic acid [21]. Apart from its antibacterial [22,23,24], antifungal [25], anti-inflammatory [26], and anthelmintic [27] properties, BITC exhibits anticancer [28] activities, as also evidenced by preclinical cancer studies [21]. 

To our knowledge, there is no information on *T. majus* and BITC usage as antimalarials. However, a recent study by Hashimoto et al. (2023) demonstrated the in vivo antimalarial activity of another isothiocyanate, allyl isothiocyanate (AITC), and its metabolite, N-acetyl-S-(N-allyl thiocarbamoyl)-l-cysteine (NAC-AITC), by in vitro and in vivo assays, both extracted from *Wasabia japonica* [29]. Arianie et al. (2021) designed novel isothiocyanates based on eugenol and cinnamaldehyde derivatives and rhamnosyloxy benzyl isothiocyanate from *Moringa oleifera* leaves, used in traditional medicine to treat malaria, by molecular docking and demonstrated their potential as antimalarials through in silico approaches [30,31].

Also, and from another perspective, in a recent study by Flor-Weiler et al. (2023) that evaluated several *Brassicaceae* seed meals as sources of plant-derived isothiocyanates, BITC displayed high larvicidal activity against *Aedes aegypti* [32].

In this study, we aimed to evaluate the antimalarial activity by flow cytometry of *T. majus* seed, leaf, and stem aqueous extracts, traditionally applied as antimicrobials, and BITC, the major biologically active compound derived from the *T. majus* parts. This assessment prompted a subsequent investigation of BITC’s cross-resistance with aminoquinolines.

## 2. Results

### 2.1. Antimalarial Screening Assessment

Flow cytometry was first used to evaluate the aqueous extracts and BITC for antimalarial activity against the asexual blood stage GFP-expressing *P. falciparum* (3D7 − GFP), a chloroquine-sensitive strain. Using the 3D7 − GFP strain obviates any staining procedure with a fluorescent dye since the parasites are auto-fluorescent, simplifying culture procedures [33,34]. The number of fluorescent events after drug exposure detected by flow cytometry, i.e., the percentage of surviving GFP parasites, allows for the determination of the growth inhibition percentage, as described in Teixeira de Morais Gomes et al. (2020).

This preliminary screening allows us to glimpse the behavior of the potential antimalarials, as well as a definition of the intervals of the concentrations to be used in the dose–response evaluation, i.e., an IC50 determination. Also, a cutoff value of 70% growth inhibition allows us to have a refined selection of potential antimalarials that can be assessed in the dose–response evaluation. Activity was then evaluated after 72 h [35,36]. The results were obtained from at least two experiments, each in triplicate, and are presented in Table 1. The DMSO percentages of 0.4% and 0.04% are the correspondent solvent concentrations on the BITC at 3.32 µM and 0.332 µM, respectively. The distilled water percentages of 1% and 0.1% are the correspondent solvent concentrations of the highest and lowest aqueous extracts concentrations, respectively (Table 1).

The differences in the concentrations of the aqueous extracts are related to the fact that, at the time of the extraction process, conserved plant materials with different weights were not available in nature, so conserved lyophilized batches were used. Given that the extracts were dissolved in distilled water, a hypotonic solvent, caution was warranted in antimalarial assays. Therefore, as an alternative approach, we opted to normalize the distilled water content rather than the concentration of the extracts by establishing two percentages, 1% and 0.1%.

The *T. majus* seed extract displayed a similar growth inhibition percentage for the tested concentrations (38.62 ± 22.89% at 132 µg/mL and 30.18 ± 13.47% at 13.2 µg/mL) and a higher growth inhibition percentage than the remaining extracts in all concentrations. The extract solvent had no antiplasmodial action at any concentration. Despite the better performance of the *T. majus* seed extract, none of the extracts presented more than 70% growth inhibition. 

Regarding benzyl isothiocyanate (BITC), at 3.32 µM, it displayed a growth inhibition above 70% (97.13 ± 0.62%) and was considered for antiplasmodial activity refinement by IC50 determination. BITC solvent (DMSO) did not show a growth inhibition percentage in both concentrations (0.4% and 0.04%). DMSO at 0.4% or 0.04% did not show a significant impact on *P. falciparum* survival and multiplication under culture conditions, corroborating previous observations [37]; hence, we proceeded with IC50 determination in the presence of 0.4% or 0.04% of DMSO (Table 1). The IC50 is a quantitative measure that allows us to evaluate *P. falciparum* growth in the presence of different concentrations by serial dilution of a compound. In this case, by diluting BITC, we also dilute the solvent, DMSO, to a much lower concentration than 0.4%.

The reference antimalarial drug chloroquine (CQ) at 10 µM and 1 µM exhibited growth inhibition percentages of 96.57 ± 0.57% and 94.71 ± 2.78%, respectively (Table 1). BITC at 3.32 µM exhibited comparable growth inhibition against *P. falciparum* to CQ at 10 µM and 1 µM.

### 2.2. Dose–Response Evaluation

BITC was considered for further analysis; dose–response curves to determine the parasite growth were evaluated using flow cytometry, and dose–response curves were determined. The half-maximal inhibitory concentration (IC50) of BITC against *P. falciparum* was calculated against strains 3D7 − GFP and Dd2, chloroquine-susceptible and chloroquine-resistant strains, respectively. 

In other words, since BITC was not tested in previous studies for its potential as an antimalarial drug, we decided to use those concentrations based on the growth inhibition assay we made before determining the IC50. The rationale of the growth inhibition assay is to provide information on the concentrations to be used in the IC50 assay and as a primary screening of the potential antimalarials. Since BITC at 3.32 µM presented a high growth inhibition percentage (above 70%), it was defined as the maximal dose to be used in the IC50 assay; BITC at 0.332 µM still presented growth inhibition, meaning that the minimal value of the BITC concentration to be used in the IC50 assay needs to be lower than 0.332 µM. Regarding CQ, since both tested concentrations had a high growth inhibition percentage, a much lower minimal concentration needed to be used in the IC50 assay, so we could have a dose–response curve.

**Figure 1:**
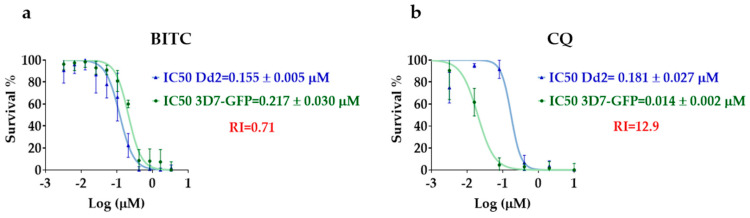
BITC biological antimalarial activity against resistant (Dd2) and susceptible (3D7 − GFP) *P. falciparum* strains. (**a**) Dose–response curves of BITC with the respective IC50 values and resistance index (RI = IC50 Dd2/IC50 3D7 − GFP); (**b**) dose–response curves of the reference drug CQ with the respective IC50 values and resistance index. The green curves correspond to the susceptible strain (3D7 − GFP), while the blue curves represent the resistant strain (Dd2). BITC, benzyl isothiocyanate; CQ, chloroquine; RI, resistance index.

The results are demonstrated in Figure 1 and were obtained from at least two experiments, each in triplicate. 

Both BITC and the reference drug CQ displayed sigmoidal dose–response curves compatible with biological activity (Figure 1). The IC50 values and the resistance index calculated for CQ were consistent with previous results in similar laboratory conditions [38,39,40], hence validating the assay conditions. The resistance index provides a quantitative measure of activity against a resistant strain in comparison to a susceptible strain (RI = IC50 Dd2/IC50 3D7 − GFP) [41,42].

The biological activity of BITC was similar against both susceptible and resistant-strains (*p* > 0.05; unpaired *t*-test), hence the absence of a shift to the right of the *P. falciparum* Dd2 dose–response curve (Figure 1) and a low resistance index (RI = 0.71). As expected, the reference drug CQ displayed lower biological activity against the resistant strain, which was significantly different (*p* < 0.05; unpaired *t*-test), hence the shift to the right of the *P. falciparum* Dd2 dose–response curve (Figure 1), i.e., an increase in the IC50 value, and a higher resistance index (RI = 12.9).

## 3. Discussion

When it comes to *T. majus* aqueous extracts, the differences in the growth inhibition percentages between the seed extract and the others can be attributable to a variety of factors.

BITC will only be free after the crushing of the material and hydrolysis of benzyl glucosinolate by endogenous enzyme myrosinase in experimental conditions. To maximize the benzyl glucosinolate extraction without inactivating the myrosinase, we used aqueous extraction at room temperature instead of organic solvents. Also, we wanted to extract benzyl glucosinolate with less income of other compounds, such as alkaloids, flavonoids and tannins, that are present in methanolic extracts and tannins and steroids present in ethanolic extracts [14,43].

After 12 h maceration of the plant material, all benzyl glucosinolate will be hydrolyzed by myrosinase at a neutral pH [44]. The levels of BITC extracted by this method in leaves and stems were not sufficient to display activity, but, in seeds, with higher concentrations of benzyl glucosinolate, the amount of BITC released to the extracts will also be heightened, which justifies the higher anti-malarial activity found with a much lower concentration of plant material. 

In this phase, we wanted to make a screen of the crude extract. Ideally, from an ethnobotanical point of view, the aqueous solution allows for the eventual use of the phytotherapeutic extracts directly in a curative way for malaria in areas of the globe where the extraction of BITC would be difficult. A future standardization of *T. majus* aqueous extraction, benzyl glucosinolate and BITC quantification of concentration levels in extracts, with GC-MS and HPLC-MS, will be pursued. For secondary metabolites, especially compounds related to stress responses like benzyl glucosinolate, plant contents are variable in terms of local origin, soil, weather, and biotic factors. Also, the freezing, extraction, and hydrolysis processes influence BITC release and content, hence the importance of the standardization conditions. 

Regarding the dose–response evaluation, the RI of BITC revealed that its phenotypic response was different than the reference drug CQ, i.e., if a compound exhibits a resistance mechanism similar to that of other antimalarials [41,42]. Since CQ is an aminoquinoline, our results suggest that BITC does not share the same resistance mechanism as aminoquinolines, which can be considered an advantage for antimalarial drug development. 

The mechanism of action of CQ is linked to its diffusion through biological membranes and concentration inside the parasite’s food vacuole, which has an acidic pH (in contrast with the neutral pH of the cytosol) [45,46]. Resistance to aminoquinolines is related to mutations in various proteins, including the transporters *P. falciparum* chloroquine resistance transporter (*Pf*CRT) and the *P. falciparum* multidrug resistance 1 protein (*Pf*MDR1) [47,48]. Since *P. falciparum* 3D7 − GFP is a CQ-sensitive strain and *P. falciparum* Dd2 a CQ-resistant strain and considering that the IC50 of BITC for both strains was identical, this strongly suggests that BITC does not have the same mechanism of action and resistance as CQ. 

Isothiocyanates have been shown to interact mainly with thiol groups, forming labile dithiocarbamate derivatives, and with amine groups, which may result in increased oxidation, i.e., the production of reactive oxygen species (ROS), and inhibition of key enzymes and/or proteins in microorganisms [49,50,51]. 

The electrophilic properties of BITC can render a high affinity for cellular sulfhydryl groups, such as enzymes and/or proteins, with functional or structural cysteine residues [23,49]. One of *P. falciparum’s* most important enzymes involved in the redox equilibrium is glutathione reductase (GR), which has cysteine residues [52,53,54]. GR is an antioxidant enzyme that catalyzes the regeneration of reduced glutathione (GSH), the active form of glutathione, from oxidized glutathione using NADPH as the source of reducing equivalents [53,54]. This reaction helps to avoid the synthesis of hydroxyl radical •OH from H_2_O_2_ produced due to hemoglobin digestion (inside the parasite’s food vacuole) and mitochondrial electron chain reactions [52,54]. A study conducted by Li et al. (2020) characterized BITC as a potential GR inhibitor in human cancer cells and demonstrated that BITC was evaluated as a competitive and irreversible GR inhibitor in a time- and concentration-dependent manner, and this reaction depended on the presence of NADPH [55]. 

Also, BITC might interfere with the GSH de novo synthesis in *P. falciparum*. It is known that in *P. falciparum*, GSH can be de novo biosynthesized by two enzymes, glutamylcysteine synthetase and glutathione synthetase, respectively, that require a source of amino acid precursors from the inactive form of glutathione (glutamate, cysteine, and glycine) [52,53]. In biological systems, a reaction between the inactive form of glutathione and BITC, catalyzed by glutathione-S-transferases, allows for the formation of a dithiocarbamate derivative, which is then effluxed from the cell [49]. This reaction may increase the interference of BITC with *P. falciparum* redox equilibrium. 

*T. majus* is characterized as a low-toxicity plant, with an LD50 above 5000 mg/kg in an in vivo mice acute toxicity study of oral administration by Zanetti et al. (2003). The absence of toxicity signs in oral administration, up to 2 weeks, could be due to the low concentrations of glucosinolates present in the extracts or due to their metabolization in the organism [56]. 

The relative toxicity of BITC in normal cells was not investigated in this work; however, BITC’s biological activity and toxicity testing on healthy mammalian cells has already been carried out in various anticancer studies, though not for malaria. BITC inhibited cell growth, promoted G2/M phase arrest, and triggered apoptosis of oral cancer OC2 cells, with minimal toxicity to normal cells [57]. Similarly, BITC has been found to induce apoptosis in breast cancer cells but has no effect on normal breast MCF-10A cells [58]. These studies suggested that BITC has selective toxicity to tumor cells and is safe to use for cancer treatment [57,58,59]. BITC can effectively exert anticancer effects at concentrations (in vitro) or doses in vivo (in vivo animals) that are no-toxic to normal tissues. With no observed adverse effect level of 50 mg/kg, which is equivalent to human consumption of around 400–570 mg daily, the BITC is considered safe to consume [21]. Also, the results of in vivo mice toxicology experiments [28] showed that animals had no evidence of major drug-induced toxicity at doses up to 100 mg/kg, with an LD50 of 140 mg/kg. Despite promising pre-clinical data, BITC has not been tested clinically for its anticancer effect [21]. Clinical studies of antimalarial activity or anticancer activity with this compound or extracts are not available yet to obtain a selectivity index. More research should be conducted to determine the therapeutic dose of BITC as a potential antimalarial.

The complexation of BITC from cyclodextrins performed by Li et al. (2015) improved the stability and aqueous solubility of this compound [60]. Hence, the antimalarial activity of the aqueous crude extracts could be improved by an enhancement in the hydrolysis of benzyl glucosinolate and solubility of BITC in water, in particular the seed extracts.

Also, the possibility of using the edible plant or its seed extracts to use in places where medicines or vaccines are not available could be an interesting line of work. Because malaria is endemic in tropical and subtropical regions of Africa, Asia, and South America, these are also regions where *T. majus* L. is adapted to grow and could easily be used as an edible plant or a source for the extraction of BITC.

These results also raise the prospect of extending the evaluation of BITC activity against other protozoan parasites. Isothiocyanates have already been shown to be active for some insects’ larvae, namely *Aedes aegypti* [32], so future work could include testing *T. majus* L and also as a plant-based bioinsecticide, enhancing a possible dual function. 

## 4. Materials and Methods

### 4.1. Plant Material

*Tropaeolum majus* L. plant material was collected in November 2022 in a cultivated field at Parque Bensaúde, Lisbon. Stems, leaves, and seeds were cleansed of residues, and the stems and leaves were also cut into small pieces. Stem, leaf, and seed FW were weighed separately and kept at −20 °C.

### 4.2. Tropaeolum majus *L.* Extraction

The extracts were prepared with previously collected, frozen, and lyophilized plant parts available with different weights. *T. majus* seeds (6.37 g *dw*), leaves (18.74 g *dw*), and stems (20 g *dw*), previously lyophilized for 72 h, were powdered with a mill and macerated in 50 mL phosphate buffer (pH 7.4) for 12 h with occasional stirring, to allow for endogenous myrosinase to promote benzyl glucosinolate degradation and benzyl isothiocyanate formation, and filtered, obtaining aqueous extracts. Afterward, the aqueous extracts were filtrated and lyophilized for 96 h, in previously tared volumetric flasks, obtaining the following 52.8 mg seed extract, 1.002 mg of leaf extract, and 527.2 mg of stem extract. Dry extracts were then dissolved in 4 mL of distilled water, obtaining the following final concentrations for the antimalarial assays: 13.2 mg/mL seed extract; 250.6 mg/mL leaf extract; 131.8 mg/mL stem extract.

### 4.3. Antimalarial Assays 

#### 4.3.1. Plasmodium Falciparum In Vitro Culture

Laboratory-adapted *P. falciparum* lines 3D7 − GFP (BEI resources, MRA-1029, MR4, ATCC^®^ Manassas, VA, USA), a chloroquine-sensitive strain, and Dd2 (cryopreserved collection from IHMT), a chloroquine-resistant strain, were continuously cultured using the modified method of Trager and Jensen [61,62]. Parasites were cultivated in 5% hematocrit, 37 °C, and an atmosphere with 5% of CO_2_ and supplemented with complete culture medium (cRPMI), as previously described [62].

#### 4.3.2. Sample Preparation

The stock solutions were made in compliance with the maximum solvent limits that can be used in antimalarial assays [37]. Keeping this in mind, a stock solution of BITC (Sigma-Aldrich^®,^ Merck KGaA, Darmstadt, Germany) with 754 µM containing 90% dimethyl sulfoxide (DMSO; Sigma-Aldrich^®^) was diluted in sterile PBS (VWRTM VWR International—Material de Laboratório, Sociedade Unipessoal Lda, Carnaxide, Portugal) to achieve a DMSO percentage in the assays ≤0.4%. The aqueous extracts were diluted in cRPMI to attain a water percentage ≤1% in the assays and previously filtrated with a 0.45-micron filter. The stock solution of the reference drug chloroquine (Sigma-Aldrich^®^) with 5000 µM containing 100% of DMSO was diluted in cRPMI to also achieve a DMSO percentage in the assays ≤0.4%. The extract solvent (distilled water), previously filtrated with a 0.45-micron filter, and BITC solvent (DMSO) were also diluted in cRPMI or sterile PBS, respectively, following the respective percentages used in the assays.

#### 4.3.3. Extracts and Compounds Screening Assessment

All aqueous extracts and compounds were screened for their in vitro antimalarial activity against *P. falciparum* 3D7 − GFP in at least two independent experiments in triplicate, as previously described, with modifications [35].

In brief, unsynchronized culture with 2% hematocrit and 1% parasitemia was incubated in a 96-well flat-bottom plate with the following concentrations for 72 h (37 °C and 5% CO_2_):

- *T. majus* seed extract: 132 µg/mL (in 1% of distilled water) and 13.2 µg/mL (in 0.1% of distilled water);
- *T. majus* leaf extract: 2510 µg/mL (in 1% of distilled water) and 251 µg/mL (in 0.1% of distilled water);
- *T. majus* stem extract: 1320 µg/mL (in 1% of distilled water) and 132 µg/mL (in 0.1% of distilled water);
- BITC: 3.32 µM: (in 0.4% of DMSO) and 0.332 µM (in 0.04% of DMSO).

Each plate also included growth control wells: untreated culture, 1%, and 0.1% of distilled water, 0.4% and 0.04% DMSO, 10 µM, and 1 µM of chloroquine (reference drug). After the incubation period, cells were diluted to achieve a 0.7% hematocrit, and the parasite growth was assessed by flow cytometry (Beckman Coulter, Cytoflex, Radnor, PA, USA) with a 96-well plate reader, using Fl-1 (green fluorescent protein (GFP); excitation wavelength, 488 nm). Typically, 100.000 RBCs were counted for each well. Samples were analyzed using FlowJo software vX 0.7 (Tree Star Inc., San Carlos, CA, USA). The growth inhibition percentage was then determined using the following formula:

(1) $$\mathrm{Growth}\;\mathrm{inhibition}\;(\%)=100-\left(\frac{Parasitemia\;treated\;culture}{\bar{X}Parasitemia\;untreated\;culture}\times 100\right)\;,$$

The extracts and/or compounds that displayed at least 70% of growth inhibition were selected as potential candidates and confirmed by IC50 estimation and resistance index determination [35].

#### 4.3.4. Dose–Response Evaluation

The antimalarial activity was estimated by previously described protocols with adjustments in at least two experiments, each in triplicate [35,36]. In short, unsynchronized cultures with 2% hematocrit and 1% parasitemia of *P. falciparum* 3D7 − GFP and Dd2 strains were incubated for 72 h (37 °C and 5% CO_2_) in a 96-well flat-bottom plate with BITC in 2-fold serial dilutions, ranging from 3.32 µM to 0.0032 µM. Additionally, each plate included growth control wells with no drug added and chloroquine as a reference drug in a 5-fold serial dilution, with concentrations ranging from 10 µM to 0.00064 µM. After 72 h, cells were diluted to achieve a 0,7% hematocrit, and the parasite growth was assessed by flow cytometry (Beckman Coulter, Cytoflex, Radnor, PA, USA) in a 96-well plate reader, using FI-1 (green; excitation wavelength, 488 nm). Before the flow cytometry reading, *P. falciparum* Dd2 strain was stained with a mixture of SYBR^TM^ Green I (Invitrogen, Thermo Fisher Scientific, Waltham, MA, USA) 0.5× in PBS 30 min in the dark in standard culture conditions. Typically, 100.000 RBCs were counted for each well. Samples were analyzed using FlowJo software vX 0.7 (Tree Star Inc., San Carlos, CA, USA). The IC50 was estimated through nonlinear regression by using the GraphPad Prism 9 software (trial version), and the resistance index was calculated using the following formula:

(2) $$\mathrm{Resistance}\;\mathrm{index}=\frac{IC50\;Dd2}{IC50\;3D7-GFP}\;,$$

A resistance index above 10 predicts a high level of resistance, whereas a resistance index below 10 might indicate an intermediate resistance level, and a resistance index close to or below 1 could reveal an absence of resistance [41]. 

#### 4.3.5. Statistical Analysis

GraphPad Prism 9 software (trial version) was used for the non-parametric Mann–Whitney test and parametric unpaired *t*-test. A significant difference was assumed when *p* < 0.05.

## 5. Conclusions

Our results on the antimalarial activity of BITC and *Tropaeolum majus* L. extracts indicate great potential for the development of new medicine. This is the first time that antimalarial activity has been recognized in this isothiocyanate, hinting at promising prospects for the development of novel antimalarials. BITC presented similar activity against both chloroquine-susceptible and -resistant *P. falciparum* strains, suggesting a different mechanism of action and, therefore, less possibility of cross-resistance with quinolines.

## Figures and Tables

**Table 1:** *P. falciparum* antimalarial screening of *T. majus* extracts, BITC, and growth controls.

Extracts, Compounds, and Growth Controls	Concentrations	*P. falciparum* Inhibition % ± SD
*T. majus* seed extract	132 µg/mL	38.62 ± 22.89 *
	13.2 µg/mL	30.18 ± 13.47 ^#^
*T. majus* leaf extract	2510 µg/mL	6.54 ± 5.32 *
	251 µg/mL	3.44 ± 2.67 ^##^
*T. majus* stem extract	1320 µg/mL	7.68 ± 3.15 *
	132 µg/mL	NI ^4^
Water (extracts solvent)	1% 0.1%	NI ^4^ NI ^4^
BITC ^1^	3.32 µM 0.332 µM	97.13 ± 0.62 14.26 ± 3.31 ^#^
DMSO ^2^ (BITC solvent)	0.4% 0.04%	NI ^4^ NI ^4^
CQ ^3^ (reference drug)	10 µM	96.57 ± 0.57
	1 µM	94.71 ± 2.78

^1^ BITC, benzyl isothiocyanate; ^2^ DMSO, dimethyl sulfoxide; ^3^ CQ, chloroquine; ^4^ NI, no inhibition observed; SD, standard deviation. Statistical analysis (Mann–Whitney and unpaired *t*-test) of results: CQ at 10 µM (* *p* < 0.05); CQ at 1 µM (# *p* < 0.05; ## *p* < 0.0001).

## Data Availability

The raw data supporting the conclusions of this article will be made available by the authors on request.
